# Fluid responsiveness in pressure support ventilation: role of asynchrony

**DOI:** 10.1186/cc12140

**Published:** 2013-03-19

**Authors:** A Messina, D Colombo, G Cammarota, M De Lucia, F Della Corte, P Navalesi

**Affiliations:** 1 Università Maggiore della Carità A. Avogadro, Novara, Italy

## Introduction

Pulse pressure variation (PPV) is a dynamic indicator of fluid responsiveness, which is known to have a low sensibility and specificity in patients ventilated in pressure support (PS) [1]. We aim to investigate patient-ventilator asynchrony as a potential source of hemodynamic interference in PS.

## Methods

We performed a prospective study including PS ventilated patients who met inclusion criteria for fluid depletion [1]. Patients who showed an asynchrony index (AI) exceeding 10% were included in the asynchrony group (AG). The remaining patients were included in the synchrony group (SG) [2]. Beat-to-beat hemodynamic variables were recorded through PRAM (Mostcare; Vytech Health srl, Padova, Italy). PPV cutoff of 13% was used to identify fluid responders/nonresponders. A fluid challenge of 500 ml normal saline was given in 5 minutes. An increase of 15% of cardiac index after 10 minutes indicated fluid responsiveness.

## Results

**Figure 1:**
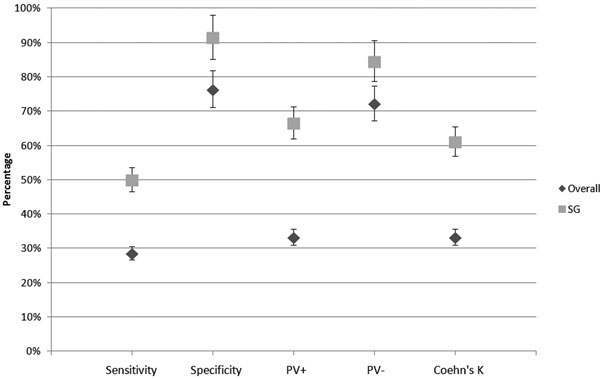


So far, eights patients showed an AI >10% while 16 did not. Overall sensitivity was 28.6% versus 50% in SG; overall specificity was 76.5% versus 91.7% in AG. Overall Cohen's *k *was 33.3% versus 61.2% in AG (see Figure 1). However, because none of the responders in the AG group was detected by PPV, statistical analysis was not feasible within this subgroup.

## Conclusion

The consistency of PPV in predicting fluid responsiveness during PS seems to be more reliable in the patients with better patient-ventilator synchrony.
